# Hot water immersion could be an effective alternative to physical exercise in improving cardiovascular fitness during the COVID-19 pandemic

**DOI:** 10.3389/fphys.2022.1035183

**Published:** 2022-11-25

**Authors:** Metodija Kjertakov, Aaron Petersen

**Affiliations:** Institute for Health and Sport, Victoria University, Melbourne, VIC, Australia

**Keywords:** passive heat exposure, hot bath treatment, peak oxygen uptake, COVID-19, general population, clinical population

Recent reports of a significant association between increased cardiorespiratory fitness (CRF) and reduced risk of developing severe coronavirus disease-2019 (COVID-19) symptoms (Brawner et al., 2021; Ekblom-Bak et al., 2021) point out the need for interventions to improve CRF in the general population. Papers published in this (Wang et al., 2020) and other journals (Dixit et al., 2020; Ranasinghe et al., 2020; Rodríguez et al., 2020; Sá Filho et al., 2020; Khoramipour et al., 2021) have already provided exercise recommendations for increasing and maintaining CRF during the COVID-19 pandemic. However, none of those papers have recognised that there are individuals who, due to temporary or permanent disabilities, are unable to exercise. Consequently, no alternative strategies to physical exercise have been provided. This paper intends to fill that gap in the COVID-19 literature.

Currently, several lines of evidence (Miyamoto et al., 2005; Ohori et al., 2012; Bailey et al., 2016; Hesketh et al., 2019) indicate that passive heat exposure may serve as an effective alternative to aerobic exercise training in improving CRF. Two of the four available studies in this research area have directly compared the effects of passive heat exposure with those of traditional exercise on CRF assessed by measuring peak oxygen uptake ( ${\dot{V}O}_{\mathrm{2peak}}$ ) during an incremental test to exhaustion administered before and after the interventions (Bailey et al., 2016; Hesketh et al., 2019). In the study by Bailey et al. (2016), 18 healthy, recreationally active, females ( ${\dot{V}O}_{\mathrm{2peak}}$ = ∼36 ml·kg^−1^·min^−1^) performed 8 weeks of moderate-intensity cycling training (*n* = 9) or hot (42 °C) water immersion to the sternum (*n* = 9) for 30 min three times per week. Interestingly, the magnitude of increase in the ${\dot{V}O}_{\mathrm{2peak}}$ value post-interventions was almost identical between the groups (∼5.5%). Hesketh et al. (2019) allocated 20 sedentary males ( ${\dot{V}O}_{\mathrm{2peak}}$ = 46 ml·kg^−1^·min^−1^) to 6 weeks of either resting in a heat chamber at 40°C for 40–50 min, three times per week (*n* = 10) or time-matched moderate-intensity cycling training (*n* = 10). Following the interventions, the passive heat exposure group experienced an increase in ${\dot{V}O}_{\mathrm{2peak}}$ comparable (5%) to that of the exercise training group (7%). Although the other two studies (Miyamoto et al., 2005; Ohori et al., 2012) did not include an exercise group, their findings confirm the cardiovascular benefits of passive heating in a clinical population. Indeed, Miyamoto et al. (2005) reported improved exercise tolerance by 15% in 15 hospitalised patients with chronic systolic congestive failure following a heating treatment consisting of 5 weekly 15–20 min sauna (60°C) bathing sessions performed over 4 weeks. In 5 of those patients, the researchers also administered a ${\dot{V}O}_{\mathrm{2peak}}$ test and observed an increase in their post-intervention ${\dot{V}O}_{\mathrm{2peak}}$ by 22%. The study by Ohori et al. (2012) recruited 41 patients with chronic heart failure to examine the effects of sitting in a 60 °C sauna room for 15 min, five times a week, for 3 weeks on exercise tolerance (in all patients) and ${\dot{V}O}_{\mathrm{2peak}}$ (in 20 patients). The heating therapy improved exercise tolerance by 12% and increased ${\dot{V}O}_{\mathrm{2peak}}$ by 8%.

In addition to the beneficial effects on CRF, there is also evidence that passive heating may improve skeletal muscle contractile function. In a study conducted on 14 healthy males, Racinais et al. (2017) found increased peak twitch amplitude and maximal voluntary torque of the soleus muscle following 11 consecutive days of whole-body heat exposure (1 h per day) in a heat chamber at 48–50°C. Given that weak muscle strength may predispose a person to severe COVID-19 (Cheval et al., 2021), the findings of Racinais et al. (2017) are highly relevant in the context of strengthening the resilience to severe forms of the disease in individuals unable to exercise. Other documented exercise mimetic properties of passive heat treatment in the form of sauna bathing and hot water immersion include reduced body weight, improved glycemic control in people with type 2 diabetes mellitus, reduced depression, and improved appetite, sleep quality, and wellbeing (Dorsey et al., 1996; Hooper et al., 1999; Naumann et al., 2017; Hayashi et al., 2022).

Obviously, of the heating methods mentioned in the two preceding paragraphs, hot water immersion has the highest practical value because many households possess the necessary equipment (i.e., a bathtub and hot water) for its implementation. Furthermore, hot water immersion is, in general, deemed safe (Thompson et al., 2017). Although heat illness has been pointed out by some as a potential consequence of taking a hot bath (Hoekstra et al., 2020), no ill health effects were observed in studies that had participants with impaired thermoregulatory capacity (i.e., spinal cord injury and diabetic patients, and elderly people) submerged up to the nipple line/neck in 39°C–42°C water for 20–60 min (Hooper et al., 1999; Gass et al., 2001; Rivas et al., 2016; Akerman et al., 2019; Yamashiro et al., 2020; James et al., 2021). Theoretically, the risk for heat stroke associated with the hot water immersion treatment described by Bailey et al. (2016) previously in the text is low because this treatment induces an increase in body core temperature no higher than 38°C. The onset of heat stroke is associated with core temperatures above 40°C (Costrini et al., 1979; Aarseth et al., 1986; Epstein et al., 1995; Kjertakov and Epstein, 2013). Available evidence indicates that hot water immersion is generally only contraindicated in people with epilepsy, as in some of this population hot bathing can provoke seizures (Stensman and Ursing, 1971; Satishchandra et al., 1988; Bebek 2001; Yalçın et al., 2006). It also needs to be noted that hot water immersion may cause transient symptomatic hypotension in some individuals (Turner et al., 1980).

Based on the findings of Bailey et al. (2016), sitting three times a week for 30 min in a bath filled with 42°C water up to the sternum (arms outside the water) for 2 months is expected to improve CRF in individuals with low fitness levels (i.e., ${\dot{V}O}_{\mathrm{2peak}}$ <50 ml·kg^−1^·min^−1^). Other possible beneficial effects of this treatment may include enhanced muscle contractility and improved mental health. However, it is unknown whether maintaining the same frequency and duration of the hot water immersion sessions beyond the 2 months treatment period will keep improving CRF. Based on the principles of adaptation theory (Adolph, 1955), one may assume that longer or more frequent hot water immersion sessions would be required to achieve further improvement in CRF. Nevertheless, long-term hot water immersion studies are needed to confirm that assumption. Currently, there is sufficient evidence to suggest that passive heating could be an effective alternative to physical exercise in improving CRF in people who cannot exercise, with hot water immersion being the most practical existing heating method.

## References

[B1] Aarseth H. P. Eide I. Skeie B. Thaulow E. (1986). Heat stroke in endurance exercise. Acta Med. Scand. 220 (3), 279–283. 10.1111/j.0954-6820.1986.tb02764.x 3535401

[B2] Adolph E. F. (1955). General and specific characteristics of physiological adaptations. Am. J. Physiol. 184 (1), 18–28. 10.1152/ajplegacy.1955.184.1.18 13283084

[B3] Akerman A. P. Thomas K. N. van Rij A. M. Body E. D. Alfadhel M. Cotter J. D. (2019). Heat therapy vs. supervised exercise therapy for peripheral arterial disease: A 12-wk randomized, controlled trial. Am. J. Physiol. Heart Circ. Physiol. 316 (6), H1495–H1506. 10.1152/ajpheart.00151.2019 31002283

[B4] Bailey T. G. Cable N. T. Miller G. D. Sprung V. S. Low D. A. Jones H. (2016). Repeated warm water immersion induces similar cerebrovascular adaptations to 8 weeks of moderate-intensity exercise training in females. Int. J. Sports Med. 37 (10), 757–765. 10.1055/s-0042-106899 27286178

[B5] Bebek N. Gürses C. Gokyigit A. Baykan B. Ozkara C. Dervent A. (2001). Hot water epilepsy: Clinical and electrophysiologic findings based on 21 cases. Epilepsia 42 (9), 1180–1184. 10.1046/j.1528-1157.2001.31000.x 11580768

[B6] Brawner C. A. Ehrman J. K. Bole S. Kerrigan D. J. Parikh S. S. Lewis B. K. (2021). Inverse relationship of maximal exercise capacity to hospitalization secondary to coronavirus disease 2019. Mayo Clin. Proc. 96 (1), 32–39. 10.1016/j.mayocp.2020.10.003 33413833 PMC7547590

[B7] Cheval B. Sieber S. Maltagliati S. Millet G. P. Formánek T. Chalabaev A. (2021). Muscle strength is associated with COVID‐19 hospitalization in adults 50 years of age or older. J. Cachexia Sarcopenia Muscle 12 (5), 1136–1143. 10.1002/jcsm.12738 34363345 PMC8426913

[B8] Costrini A. M. Pitt H. A. Gustafson A. B. Uddin D. E. (1979). Cardiovascular and metabolic manifestations of heat stroke and severe heat exhaustion. Am. J. Med. 66 (2), 296–302. 10.1016/0002-9343(79)90548-5 425971

[B9] Dixit S. (2020). Can moderate intensity aerobic exercise be an effective and valuable therapy in preventing and controlling the pandemic of COVID-19? Med. Hypotheses 143, 109854. 10.1016/j.mehy.2020.109854 32464492 PMC7237357

[B10] Dorsey C. M. Lukas S. E. Teicher M. H. Harper D. Winkelman J. W. Cunningham S. L. (1996). Effects of passive body heating on the sleep of older female insomniacs. J. Geriatr. Psychiatry Neurol. 9 (2), 83–90. 10.1177/089198879600900203 8736588

[B11] Ekblom-Bak E. Väisänen D. Ekblom B. Blom V. Kallings L. V. Hemmingsson E. (2021). Cardiorespiratory fitness and lifestyle on severe COVID-19 risk in 279, 455 adults: A case control study. Int. J. Behav. Nutr. Phys. Act. 18 (1), 135. 10.1186/s12966-021-01198-5 34666788 PMC8524225

[B12] Epstein Y. Sohar E. Shapiro Y. (1995). Exertional heatstroke: A preventable condition. Isr. J. Med. Sci. 31 (7), 454–462. 7607879

[B13] Gass E. M. Gass G. C. (2001). Thermoregulatory responses to repeated warm water immersion in subjects who are paraplegic. Spinal Cord. 39 (3), 149–155. 10.1038/sj.sc.3101117 11326325

[B14] Hayashi E. Aoyama M. Fukano F. Takano J. Shimizu Y. Miyashita M. (2022). Effects of bathing in a tub on physical and psychological symptoms of end-of-life cancer patients: An observational, controlled study. J. Hosp. Palliat. Nurs. 24 (1), 30–39. 10.1097/NJH.0000000000000803 34550913 PMC8728761

[B15] Hesketh K. Shepherd S. O. Strauss J. A. Low D. A. Cooper R. J. Wagenmakers A. J. (2019). Passive heat therapy in sedentary humans increases skeletal muscle capillarization and eNOS content but not mitochondrial density or GLUT4 content. Am. J. Physiol. Heart Circ. Physiol. 317 (1), H114–H123. 10.1152/ajpheart.00816.2018 31074654

[B16] Hoekstra S. Bishop N. Leicht C. (2020). Elevating body temperature to reduce low-grade inflammation: A welcome strategy for those unable to exercise? Exerc. Immunol. Rev. 26, 42–55. 32139348

[B17] Hooper P. L. (1999). Hot-tub therapy for type 2 diabetes mellitus. N. Engl. J. Med. 341 (12), 924–925. 10.1056/NEJM199909163411216 10498473

[B18] James T. J. Corbett J. Cummings M. Allard S. Young J. S. Towse J. (2021). Timing of acute passive heating on glucose tolerance and blood pressure in people with type 2 diabetes: A randomized, balanced crossover, control trial. J. Appl. Physiol. 130 (4), 1093–1105. 10.1152/japplphysiol.00747.2020 33411640

[B19] Jiménez-Pavón D. Carbonell-Baeza A. Lavie C. J. (2020). Physical exercise as therapy to fight against the mental and physical consequences of COVID-19 quarantine: Special focus in older people. Prog. Cardiovasc. Dis. 63 (3), 386–388. 10.1016/j.pcad.2020.03.009 32220590 PMC7118448

[B20] Khoramipour K. Basereh A. Hekmatikar A. A. Castell L. Ruhee R. T. Suzuki K. (2021). Physical activity and nutrition guidelines to help with the fight against COVID-19. J. Sports Sci. 39 (1), 101–107. 10.1080/02640414.2020.1807089 32842905

[B21] Kjertakov M. Epstein Y. (2013). Exertional heat stroke in athletes. Maced. J. Med. Sci. 1 (1), 473–477. 10.3889/mjms.1857-5773.2013.0308

[B22] Miyamoto H. Kai H. Nakaura H. Osada K. Mizuta Y. Matsumoto A. (2005). Safety and efficacy of repeated sauna bathing in patients with chronic systolic heart failure: A preliminary report. J. Card. Fail. 11 (6), 432–436. 10.1016/j.cardfail.2005.03.004 16105634

[B23] Naumann J. Grebe J. Kaifel S. Weinert T. Sadaghiani C. Huber R. (2017). Effects of hyperthermic baths on depression, sleep and heart rate variability in patients with depressive disorder: A randomized clinical pilot trial. BMC Complement. Altern. Med. 17 (1), 172–179. 10.1186/s12906-017-1676-5 28351399 PMC5371197

[B24] Ohori T. Nozawa T. Ihori H. Shida T. Sobajima M. Matsuki A. (2012). Effect of repeated sauna treatment on exercise tolerance and endothelial function in patients with chronic heart failure. Am. J. Cardiol. 109 (1), 100–104. 10.1016/j.amjcard.2011.08.014 21944673

[B25] Racinais S. Wilson M. G. Périard J. D. (2017). Passive heat acclimation improves skeletal muscle contractility in humans. Am. J. Physiol. Regul. Integr. Comp. Physiol. 312 (1), R101-R107–R107. 10.1152/ajpregu.00431.2016 27903515

[B26] Ranasinghe C. Ozemek C. Arena R. (2020). Exercise and well-being during COVID 19–time to boost your immunity. Expert Rev. anti. Infect. Ther. 18 (12), 1195–1200. 10.1080/14787210.2020.1794818 32662717

[B27] Rivas E. Newmire D. E. Ben-Ezra V. (2016). Obese type 2 diabetics have a blunted hypotensive response to acute hyperthermia therapy that does not affect the perception of thermal stress or physiological strain compared to healthy adults. Physiol. Behav. 165, 374–382. 10.1016/j.physbeh.2016.08.026 27570191

[B28] Rodríguez M. Á. Crespo I. Olmedillas H. (2020). Exercising in times of COVID-19: What do experts recommend doing within four walls? Rev. Esp. Cardiol. 73 (7), 527–529. 10.1016/j.recesp.2020.04.002 PMC7142674 32414660

[B29] Sá Filho A. S. Miranda T. G. de Paula C. C. Barsanulfo S. R. Teixeira D. Monteiro D. (2020). COVID-19 and quarantine: Expanding understanding of how to stay physically active at home. Front. Psychol. 11, 566032. 10.3389/fpsyg.2020.566032 33192841 PMC7658189

[B30] Satishchandra P. Shivaramakrishana A. Kaliaperumal V. G. Schoenberg B. S. (1988). Hot water epilepsy: A variant of reflex epilepsy in southern India. Epilepsia 29 (1), 52–56. 10.1111/j.1528-1157.1988.tb05098.x 3338422

[B31] Stensman R. Ursing B. (1971). Epilepsy precipitated by hot water immersion. Neurology 21 (5), 559–562. 10.1212/WNL.21.5.559 5104101

[B32] Thompson K. M. Coates A. M. Incognito A. V. Whinton A. K. (2017). Chronic heat exposure for health and exercise performance–cardiovascular research heats up. J. Physiol. 595 (13), 4137–4138. 10.1113/JP274003 28390060 PMC5491892

[B33] Turner B. Pennefather J. Edmonds C. (1980). Cardiovascular effects of hot water immersion (suicide soup). Med. J. Aust. 2 (1), 39–40. 10.5694/j.1326-5377.1980.tb131813.x 7432266

[B34] Wang M. Baker J. S. Quan W. Shen S. Fekete G. Gu Y. (2020). A preventive role of exercise across the coronavirus 2 (SARS-CoV-2) pandemic. Front. Physiol. 11, 572718. 10.3389/fphys.2020.572718 33013486 PMC7506115

[B35] Yalçın A. D. Toydemir H. E. Forta H. (2006). Hot water epilepsy: Clinical and electroencephalographic features of 25 cases. Epilepsy Behav. 9 (1), 89–94. 10.1016/j.yebeh.2006.03.013 16698323

[B36] Yamashiro M. Nishimura Y. Mikami Y. Kouda K. Sakurai Y. Yoshioka I. (2020). Attenuation of core temperature elevation and interleukin-6 excretion during head-out hot water immersion in elderly people. J. Phys. Ther. Sci. 32 (7), 444–448. 10.1589/jpts.32.444 32753784 PMC7344289

